# Associations of economic vulnerability and food insecurity with the Planetary Health Diet in children: PASE study (Brazil)

**DOI:** 10.3389/fnut.2025.1706243

**Published:** 2026-02-13

**Authors:** Érica Priulli, Mariana de Santis Filgueiras, Dayane de Castro Morais, Bruna Clemente Cota, Juliana Farias de Novaes

**Affiliations:** Department of Nutrition and Health, Universidade Federal de Viçosa, Viçosa, Minas Gerais, Brazil

**Keywords:** children, food insecurity, Planetary Health Diet, social vulnerability, sustainable diet

## Abstract

**Background/objectives:**

This study investigated the associations of economic vulnerability and food insecurity (FI) with the Planetary Health Diet (PHD).

**Methods:**

This cross-sectional study included 378 children aged 8 to 9 years from Viçosa, Minas Gerais, Brazil. Food consumption was assessed using 24-h dietary recalls, and adherence to the PHD was measured using the Planetary Health Diet Index (PHDI). Child and family sociodemographic characteristics were assessed using a semi-structured questionnaire. Food insecurity was evaluated using the Brazilian Food Insecurity Scale. Data were analyzed using adjusted linear regression models.

**Results:**

The mean PHDI score was low (37.5), indicating limited overall alignment with the PHD recommendations. Higher relative alignment with the PHDI scoring criteria was observed among children from more economically vulnerable households, including those living in poverty (≤US$5.50 per capita/day), with moderate or severe food insecurity, receiving government assistance, and in households with a higher number of residents. Moreover, poverty was associated with reduced consumption of animal-based foods, whole cereals, fruits, red and orange vegetables, and increased intake of affordable staples such as legumes and vegetable oils.

**Conclusion:**

While children in economically vulnerable households appeared to follow dietary patterns more relatively aligned with the PHD, it seems to be incidental, and likely reflects financial constraints due to economic hardship rather than active sustainability choices. Policymakers should interpret such adherence cautiously and prioritize equitable access to diverse, nutritious foods that align with both health and environmental goals. Further studies are needed to clarify how socioeconomic disparities shape relative adherence to the PHD.

## 1 Introduction

Moderate or severe food insecurity (FI) affects approximately one-third of the global population (1), contributing to the “lack of secure access to sufficient amounts of safe and nutritious food for normal growth development and an active and healthy life” (2). As an aggravating factor, countries with the highest prevalence of FI are also those most affected by the impacts of climate change, which further exacerbates economic and nutritional inequalities (3).

In this context, the United Nations Sustainable Development Goals (SDGs) set several targets, such as achieving food security and promoting sustainable agriculture (SDG 2), reducing inequality within and among countries (SDG 10), and taking action to combat climate change and its impacts (SDG 13) (4). Moreover, the promotion of healthy and sustainable diets is recommended to reduce childhood malnutrition (5) and contribute to the achievement of the SDGs, as it remains largely overlooked in global policy agendas (6). Beyond environmental benefits, sustainable diets are associated with higher overall diet quality and lower consumption of ultra-processed foods (7, 8). However, socioeconomic constraints can limit access to nutrient-rich foods, making such diets inaccessible for vulnerable economic populations (9).

Based on the concept of planetary health, which encompasses human health and the preservation of natural systems on which it depends, the EAT-Lancet Commission proposed the Planetary Health Diet (PHD) as part of transforming food systems to achieve the SDGs. By 2050, combined with other sustainable practices, the PHD could feed up to 10 billion people within planetary boundaries (10). Developed in Brazil, the Planetary Health Diet Index (PHDI) assesses adherence to these recommendations (7), however, to our knowledge, no study has evaluated adherence to the PHD focused on Brazilian children.

The cost of the PHD varies across countries and, even if generally lower in low-income countries, may remain inaccessible in economically vulnerable settings, as is estimated that the cost for adhering the PHD diet exceeded household per capita income for at least 1.58 billion people, which the largest share in the cost of fruits and vegetables, followed by legumes and nuts, meat, eggs, and fish, and dairy (9). The aim of the study was to evaluate the associations of economic vulnerability and food insecurity with adherence to the PHD among Brazilian children, with the hypothesis that greater economic vulnerability would be associated with lower adherence due the higher cost of healthy foods.

## 2 Materials and methods

### 2.1 Study design and participants

This cross-sectional study is part of the “Schoolchildren Health Assessment Survey” (*Pesquisa de Avaliação da Saúde do Escolar − PASE*, in Portuguese), conducted with children aged 8 and 9 years enrolled in all urban schools (17 public and 7 private) in Viçosa, Minas Gerais, Brazil. Viçosa is a predominantly urban municipality with approximately 72,000 inhabitants, located 227 km from the state capital, Belo Horizonte (11).

The sample size calculation and sampling procedures have been described in detail in previous publications (12). Briefly, in 2015, a sample of 378 children aged 8 and 9 years was randomly selected from a population of 1,464 children in this age group enrolled in all public and private urban schools. Children with health conditions affecting nutritional status or body composition, those chronically using medications that interfere with glucose and/or lipid metabolism, and those for whom guardians could not be successfully contacted after three attempts were not included in the study.

All participant data were treated with strict confidentiality. Personal identifiers were removed during data entry, and each participant was assigned a unique identification code to ensure anonymity. Electronic data were stored in password-protected databases, with access restricted to authorized members of the research team. All results are presented in aggregate form to prevent the identification of individual participants.

This investigation was conducted in accordance with the guidelines established by the Declaration of Helsinki and approved by the Human Research Ethics Committee of the *Universidade Federal de Viçosa (UFV)* (no. 663.171/2014). Before beginning participation, children received verbal explanations in age-appropriate language, and their voluntary participation was respected. Subsequently, their guardians provided a written Informed Consent Form (ICF).

### 2.2 Planetary Health Diet index (PHDI)

Food consumption was assessed using the average of three 24-h dietary recalls (24HRs), with at least 15 days between them and including one weekend day. Trained nutritionists conducted the recalls using the five-step multiple-pass method (13), with children being assisted by guardians and using household utensils and portion-size photographs to estimate portions (14). To analyze the nutritional value of foods, the Brazilian Food Composition Table (*Tabela Brasileira de Composição de Alimentos – TBCA* in Portuguese), version 7.0 (15), was used. The TBCA was developed in accordance with the guidelines of the International Network of Food Data Systems (INFOODS). To assess the plausibility of reported energy intake, extreme values beyond three times the interquartile range were considered implausible, reflecting potential under- or over-reporting (16, 17). For recipe breakdown, we utilized a database containing nutritional compositions and standardized Brazilian recipes, according to the TBCA (15). Food consumption data were initially entered into the Diet Pro® 5i software, version 5.8 (1997), and then exported to Microsoft Excel. The linkage with TBCA was performed using Power Query (18) and Visual Basic for Applications (19) extensions.

The PHDI was used to measure adherence to the Planetary Health Diet (PHD) (7). First, all mixed dishes and processed foods were disaggregated into their basic ingredients using a Brazilian database of standard recipes. For highly processed products that are mainly composed of a single base ingredient, such as those made primarily from maize starch or wheat flour, the energy contribution was estimated based on their fat and added sugar content. Processed meats were categorized based on main ingredient or marketed formulation (e.g., sausage, ham, and salami) or chicken and substitutes (e.g., nuggets) (7).

Foods were grouped into four categories and 16 PHDI food groups: Adequacy (nuts and peanuts, legumes, fruits, vegetables, and whole cereals); Optimum (eggs, dairy, fish and seafood, tubers, and vegetable oils); Ratio (the ratio of dark green vegetables and red and orange vegetables to total vegetables); and Moderation (red meat, chicken and substitutes, animal fats, and added sugars) (7). After classifying the foods, the percentage of energy contribution for each group was calculated (group calories ÷ total calories consumed × 100), and PHDI component score was calculated based on closeness to the PHD targets. For “Adequacy,” the maximum score (10 points) was given when consumption met or exceeded the target. For “Moderation,” the maximum score (10 points) corresponded to no consumption, decreasing proportionally down to the recommended minimum intake limits. For the “Optimum” (10 points) and “Ratio” (5 points) components, the maximum score was assigned when the target was reached, becoming inversely proportional as consumption exceeded the target (7). Supplementary Table 1 details the PHDI components, cut-off points and scoring criteria (8).

It should be noted that the PHDI was initially validated for use with Brazilian adults. In this study, the PHDI was applied to assess relative adherence to the PHD among children. Therefore, results should be interpreted with caution, as the findings are discussed in terms of proportional alignment with the PHD framework rather than assuming full validity of the index for children.

### 2.3 Sociodemographic variables

Trained researchers applied a semi-structured questionnaire to guardians to obtain demographic data on children (sex, age, and skin color) and indicators of economic vulnerability (maternal education, per capita household income, FI, participation in government assistance programs, and household size).

The skin color of the study participants was self-reported as White, Brown, Black, or Asian, following the classification adopted by the Brazilian Institute of Geography and Statistics. Maternal education was assessed based on the number of years of schooling completed by the child’s mother. This variable was categorized into three groups: ≤4 years, 5–10 years, and ≥11 years.

The per capita household income was calculated by dividing the total monetary income of all household members by the total number of income dependents. Income was converted from Brazilian real to U.S. dollars based on the exchange rate in effect during the study year (US$1.00 = R$3.33) and analyzed according to the World Bank’s international poverty line recommendations: poverty (≤US$5.50 per day) and above the poverty line (>US$5.50 per day) (20).

The FI was evaluated through interviews with parents/guardians according to the Brazilian Household Food Insecurity Measurement Scale (*Escala Brasileira de Insegurança Alimentar − EBIA*, in Portuguese). The EBIA, validated for the Brazilian population (21) and based on the scale of the United States Department of Agriculture (22), consists of 14 questions related to the previous 3 months. This scale has cutoff points to measure FI in households with children under 18 years of age, and was categorized as: Food security (zero positive answers), Mild food insecurity (1 to 5 positive answers), and Moderate or Severe food insecurity (6 to 14 affirmative answers) (21, 29).

Participation in government assistance programs was considered when any family member received assistance, such as *Bolsa Família, Cesta de Alimentos, Programa de Erradicação do Trabalho Infantil (PETI), Assistência a Pessoas Idosas e Deficientes (BPC), Programa Nacional de Fortalecimento da Agricultura Familiar (PRONAF), Auxílio-desemprego*, or other programs. This was analyzed as a binary variable (yes/no).

The household size considered the total number of household members, regardless of the number of rooms in the home. This variable was categorized into three groups: 2–3; 4–5; and 6–8 people per household.

### 2.4 Anthropometric and body composition evaluation

Weight was obtained using a digital electronic scale with a capacity of 150 kg and sensitivity of 100 g (Tanita Ironman Model BC 553, Tanita Corporation of America Inc., Arlington Heights, IL, United States) and height was measured using a vertical stadiometer divided into centimeters and subdivided into millimeters (Alturexata, Belo Horizonte, Brazil). Based on these measurements, the Z score for BMI/age was calculated using the WHO Anthro Plus software, according to sex- and age-specific references for children (23). Body fat was estimated using dual-energy X-ray absorptiometry (DXA; Lunar Prodigy Advance, GE Medical Systems Lunar, Milwaukee, WI, EUA), and classified according to the cutoff points: excess body fat: ≥25% for females and ≥20% for males (24).

### 2.5 Statistical analysis

*Exposure*. Economic vulnerability indicators (maternal education, per capita household income, FI, participation in government assistance programs, and household size).

*Outcome*. PHDI.

*Covariables*. Age (continuous), sex, skin color, percentage of body fat (continuous), and total energy intake.

Statistical analysis were adjusted for sociodemographic characteristics (sex, age, and skin color) as determinants of inequalities of food insecurity and diet quality in Brazil (25, 26); for total energy intake, as recommended in the PHDI validation study (7); and for body fat because it is associated with economic vulnerability and diet quality (27, 28). The normality of the PHDI scores and its 16 components was assessed using the Shapiro–Wilk test. The scores were presented as mean and standard deviation (SD), and the sample characteristics as absolute and relative frequencies (%). Differences in PHDI according to sample characteristics were analyzed using Student’s *t*-test and one-way ANOVA with Tukey’s post-hoc test. Adjusted linear regression models, for each exposure representing socioeconomic vulnerability and FI, were used to analyze their association with PHDI, as well as between per capita household income and each PHDI component, considering both energy-adjusted intake and component scores, as a sensitivity analysis. Multiple linear regression models with robust standard errors were used, considering the heteroscedasticity and non-normality (29). Statistical analyses were performed using Stata 17.0 software, adopting a significance level of 5%.

## 3 Results

The study included 378 children with a mean age of 8.5 years (SD = 0.5). Child and family sociodemographic characteristics are presented in Table 1. Regarding nutritional status, 3.2% had thinness, 64% presented normal weight and 32.8% excess weight (overweight and obesity). The prevalence of excess body fat was 35.5%.

**Table 1:** Planetary Health Diet index (PHDI) according to sociodemographic characteristics and food insecurity in children (Viçosa, Minas Gerais, Brazil, 2015).

Variables	Total	PHDI	*p*-value
	*n* (%)	Mean (SD)	
Total	378 (100%)	37.5 (10.6)	
Age (years)			0.96
8	183 (48.4%)	37.4 (10.8)	
9	195 (51.6%)	37.5 (10.4)	
Sex			0.07
Female	197 (52.1%)	38.4 (10.2)	
Male	181 (47.9%)	36.4 (10.9)	
Skin color^†^			0.22
White	119 (31.5%)	36.5 (9.8)	
Brown	211 (55.8%)	37.4 (10.9)	
Black	43 (11.4%)	40.0 (10.8)	
Asian	5 (1.3%)	41.6 (7.5)	
Type of school			0.12
Public	268 (70.9%)	38.0 (10.3)	
Private	110 (29.1%)	36.1 (11.2)	
Maternal education (years)^†^			**0.026**
≤4	53 (14.0%)	39.2 (10.0)^ab^	
5–10	109 (28.8%)	39.1 (10.4)^a^	
≥11	216 (57.2%)	36.2 (10.7)^b^	
International poverty lines			**<0.001**
Poverty (≤5.50/day)	218 (57.7%)	39.0 (10.1)	
Above (>US$ 5.50/day)	160 (42.3%)	35.4 (10.9)	
Food (in)security^†^			
Food security	195 (51.6%)	36.5 (10.7)^a^	**0.046**
Mild food insecurity	145 (38.4%)	37.9 (10.6)^a^	
Moderate/severe food insecurity	38 (10.0%)	41.0 (9.4)^b^	
Assistance programs			
No	272 (72%)	36.7 (10.9)	**0.024**
Yes	106 (28%)	39.4 (9.4)	
Household size^†^			
2–3	114 (30.2%)	35.3 (10.8)^a^	**0.003**
4-5	239 (63.2%)	37.9 (10.3)^a^^b^	
6–8	25 (6.6%)	42.8 (10.1)^b^	

PHDI, Planetary Health Diet Index; SD, standard deviation. Student’s *t*-test. Values in bold (*p* < 0.05).

† One-way ANOVA test, with Tukey post-hoc. Different letters represent significant differences between groups.

**Figure 1:**
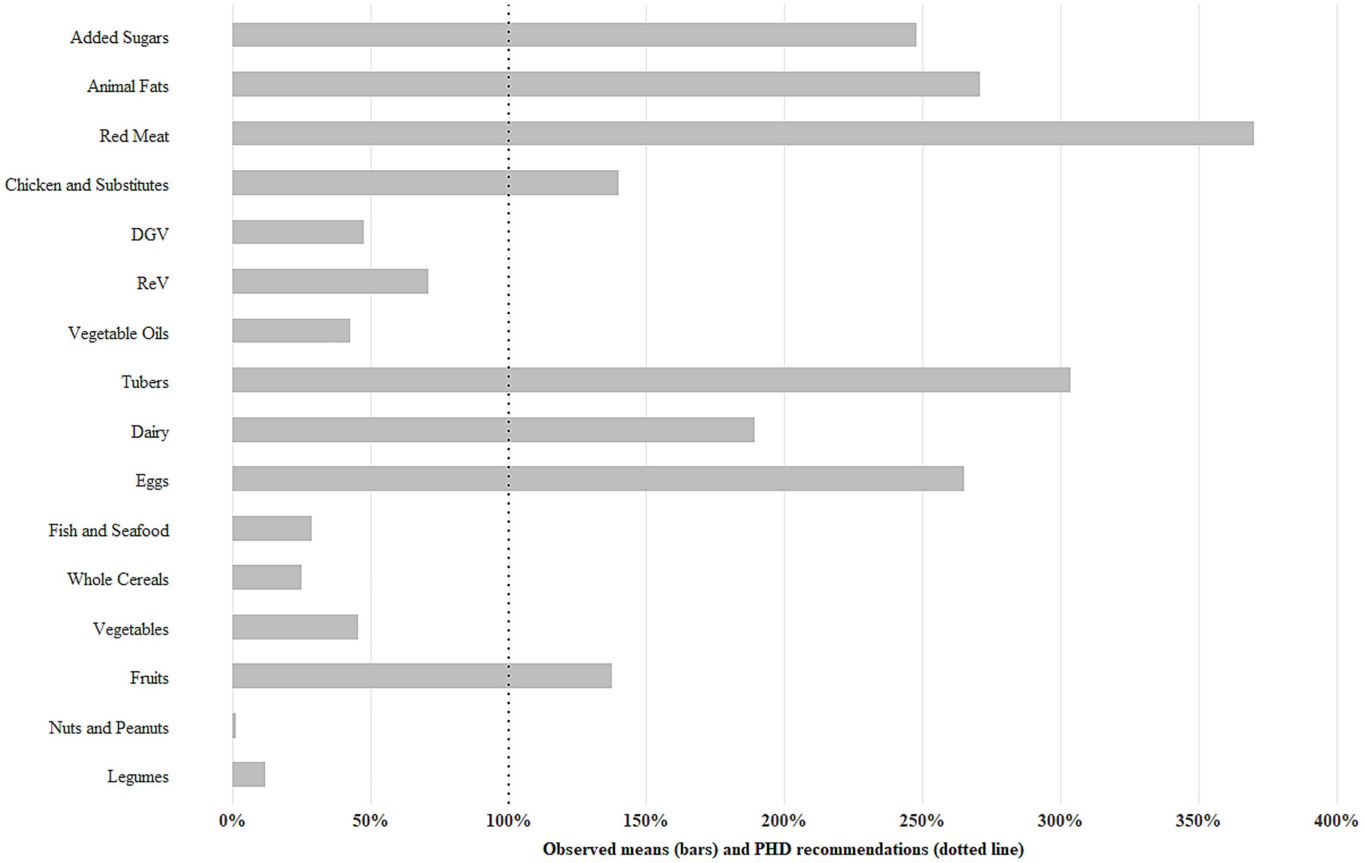
Recommendations for consumption of the Planetary Health Diet (PHD) and the observed means in children. Viçosa, Minas Gerais, Brazil, 2015. Daily energy intake recommendations were based on the Planetary Health Diet cut-off points (Supplementary Table 1): Fruits ≥5%; vegetables ≥3.1%; nuts and peanuts ≥11.6%; whole cereals ≥11.3%; legumes ≥32.4%; eggs = 0.8%; dairy = 6.1%; tubers = 1.6%; vegetable oils = 16.5%; fish and seafood = 1.6%; dark green vegetables/total vegetables (DGV) = 29.5%; red vegetables/total vegetables (ReV) = 38.5%; red meat ≤2.4%; chicken and substitutes ≤5%; animal fat ≤1.4%; added sugar ≤4.8%.

The adherence to the PHDI was assessed based on the percentage of energy intake in relation to the daily recommendation. On average, consumption exceeded the recommended levels for added sugars, animal fats, chicken and substitutes, red meat, dairy, tubers, eggs, and fruits. However, the consumption of red and orange vegetables (ReV), dark green vegetables (DGV), vegetable oils, fish and seafood, whole cereals, vegetables, legumes, nuts, and peanuts was below the target proposed by the PHDI (Figure 1).

The mean PHDI score was 37.5 (SD = 10.6), indicating limited overall relative alignment with the PHD recommendations in this sample. No child achieved the maximum PHDI recommendation of 150 points. The PHDI median was 38 (interquartile range: 30.8–44.0), and the minimum and maximum scores observed were 6.8 and 69.8, respectively.

Children with greater economic vulnerability and FI presented higher PHDI scoring criteria, including those from families with lower maternal education, living below the poverty line, with moderate or severe FI, receiving government assistance, or living in households with more residents (Table 1).

After adjusting for confounding variables, children living below the poverty line (*β* = 3.1; 95%CI: 0.6, 5.6), experiencing moderate or severe FI (*β* = 3.6; 95%CI: 0.2, 7.0), receiving government assistance (*β* = 2.5; 95%CI: 0.1, 4.9), and living in households with a greater number of residents (*β* = 1.4; 95%CI: 0.3, 2.5; p-trend = 0.010) had higher PHDI scores (Table 2).

**Table 2:** Associations of economic vulnerability and food insecurity with the Planetary Health Diet Index (PHDI) in children (Viçosa, Minas Gerais, Brazil, 2015).

Variables	Unadjusted model^†^	Model 1	Model 2
	*β* (95%CI)	*β* (95%CI)	*β* (95%CI)
Maternal education (years)			
≤4	Reference	Reference	Reference
5–10	−0.1 (−3.4; 3.2)	−0.2 (−3.6; 3.1)	0.2 (−3.1; 3.6)
≥11	**−3.0 (−6.1; −0.01)**	−3.1 (−6.2; 0.1)	−2.8 (−5.9; 0.3)
Per 1 SD (95%CI)^§^	**−1.20 (−2.27; −0.12)**	−1.15 (−2.30; −0.001)	−1.10 (−2.22; 0.01)
*p* trend^‖^	**0.029**	0.050	0.053
International poverty lines			
Above (>US$ 5.50)	Reference	Reference	Reference
Poverty (≤US$ 5.50)	**3.6 (1.5; 5.8)**	**3.6 (1.4; 5.8)**	**3.1 (0.6; 5.6)**
Per 1 SD (95%CI) ^§^	−0.30 (−1.61; 1.02)	−0.21 (−1.61; 1.18)	−0.26 (−1.64; 1.11)
*p* trend^‖^	0.66	0.77	0.71
Food (in)security			
Security	Reference	Reference	Reference
Mild insecurity	1.4 (−0.9; 3.7)	1.2 (−1.1; 3.5)	1.0 (−1.2; 3.3)
Moderate/severe insecurity	**4.5 (1.2; 7.8)**	**4.1 (0.7; 7.5)**	**3.6 (0.2; 7.0)**
Per 1 SD (95%CI) ^§^	**1.15 (0.12; 2.19)**	1.05 (−0.02; 2.13)	0.89 (−0.18; 1.95)
*p* trend^‖^	**0.029**	0.054	0.102
Assistance programs			
No	Reference	Reference	Reference
Yes	**2.73 (0.4; 5.1)**	**2.7 (0.3; 5.1)**	**2.5 (0.1; 4.9)**
*p*-value	**0.024**	**0.028**	**0.039**
Household size			
2–3	Reference	Reference	Reference
4–5	**2.7 (0.3; 5.0)**	**2.6 (0.2; 5.0)**	**2.9 (0.5; 5.3)**
6–8	**7.5 (3.2; 11.9)**	**7.2 (2.7; 11.6)**	**7.1 (2.7; 11.4)**
Per 1 SD (95%CI) ^§^	**1.6 (0.5; 2.6)**	**1.4 (0.4; 2.5)**	**1.4 (0.3; 2.5)**
*p* trend^‖^	**0.004**	**0.010**	**0.010**

FI, food insecurity; 95%CI, 95% confidence interval; SD, standard deviation. ^†^Linear regression models with indicators of economic vulnerability and food insecurity as predictors and the Planetary Health Diet Index (PHDI) as continuous outcome. Robust variance estimates were specified in all models. Model 1: adjustment for age, sex, and skin color. Model 2: Model 1 + adjustment for body fat percentage and total energy intake/day. ^§^From linear regression model with the PHDI as continuous outcome, and indicators of economic vulnerability and food insecurity per 1 SD (continuous) as predictors. ^‖^Test for linear trend when a variable representing ordinal categories of indicators of economic vulnerability and food insecurity were introduced as a continuous predictor in the linear regression. Values in bold (*p* < 0.05).

Regarding the sensitivity analyses of the PHDI components, energy intake of fruits, fish and seafood, dairy products, and ReV, as well as the component scores for fruits, whole cereals, and ReV, were positively associated with per capita income (above the poverty line). Conversely, both the energy intake and the scores for legumes and vegetable oils were inversely associated with per capita income (above the poverty line). For instance, as shown in Table 3, children below the poverty line consumed 9.8% kcal from dairy products, compared to 13.9% kcal among those above the poverty line. Likewise, for each one-standard-deviation increase in income, energy intake was approximately 1% kcal higher from dairy products, +0.4% kcal from fish, +1.6% kcal from fruits, and +3.1% kcal from red and orange vegetables (ReV), while intake from vegetable oils and legumes decreased by 1.1% kcal and 0.7%, respectively.

**Table 3:** Association between per capita income and PHDI components in children (Viçosa, Minas Gerais, Brazil, 2015).

PHDI components	Mean (SD)	Poverty line		Per 1 SD (95%CI)^§^	*p*-trend^‖^
		Yes	No		
Nuts and peanuts					
% EI	0.1 (1.1)	0.16 (1.38)	0.12 (0.68)	0.0 (−0.1; 0.1)	0.74
Score	0.1 (0.8)	0.11 (0.89)	0.10 (0.58)	0.0 (−0.1; 0.1)	0.93
Legumes					
% EI	3.9 (2.9)	4.89 (3.18)	2.53 (1.83)	**−0.7 (−1.0; −0.3)**	**<0.001**
Score	3.8 (2.4)	4.22 (2.45)	2.24 (1.62)	**−0.6 (−0.8; −0.3)**	**<0.001**
Fruits					
% EI	6.9 (6.0)	5.34 (4.90)	8.97 (6.58)	**1.6 (1.0; 2.1)**	**<0.001**
Score	7.3 (3.5)	6.73 (3.60)	8.13 (3.21)	**0.6 (0.4; 0.9)**	**0.001**
Vegetables					
% EI	1.4 (1.1)	1.52 (1.12)	1.27 (0.92)	0.0 (−0.1; 0.1)	0.45
Score	4.4 (2.5)	4.61 (2.53)	3.96 (2.49)	−0.1 (−0.4; 0.2)	0.43
Whole cereals					
% EI	2.9 (3.9)	2.36 (3.05)	3.53 (4.78)	−0.1 (−0.4; 0.2)	0.43
Score	0.9 (1.2)	0.73 (0.94)	1.09 (1.47)	**0.2 (0.1; 0.4)**	**0.003**
Eggs					
% EI	2.1 (2.4)	2.13 (2.43)	2.11 (2.38)	0.2 (−0.2; 0.5)	0.33
Score	2.3 (3.2)	2.20 (3.12)	2.46 (3.33)	0.0 (−0.3; 0.3)	0.95
Fish and seafood					
% EI	0.5 (1.6)	0.28 (1.23)	0.71 (1.88)	**0.4 (0.2; 0.7)**	**<0.001**
Score	0.7 (2.2)	0.57 (2.07)	0.87 (2.43)	0.2 (−0.1; 0.5)	0.28
Tubers					
% EI	4.9 (5.5)	4.93 (5.77)	4.75 (5.06)	0.0 (−0.5; 0.6)	0.92
Score	1.4 (2.7)	1.29 (2.66)	1.50 (2.84)	0.0 (−0.3; 0.2)	0.93
Dairy					
% EI	11.5 (6.9)	9.82 (6.02)	13.89 (7.41)	**1.0 (0.1; 1.9)**	**0.04**
Score	3.3 (5.5)	3.97 (3.61)	2.67 (3.12)	−0.1 (−0.5; 0.4)	0.79
Vegetable oils					
% EI	7.1 (4.0)	8.07 (4.06)	5.72 (3.36)	**−1.1 (−1.4; −0.7)**	**<0.001**
Score	4.2 (2.3)	4.83 (2.32)	3.44 (1.94)	**−0.6 (−0.8; −0.4)**	**<0.001**
DGV					
% EI	14.0 (20.2)	14.11 (20.01)	13.93 (20.47)	0.1 (−0.1; 0.3)	0.52
Score	1.4 (1.7)	1.46 (1.73)	1.37 (1.66)	0.2 (−1.9; 2.4)	0.82
ReV					
% EI	27.4 (20.0)	24.49 (19.21)	31.31 (20.45)	**3.1 (0.8; 5.3)**	**0.01**
Score	1.4 (1.7)	**2.52 (1.58)**	**2.91 (1.50)**	**0.2 (0.03; 0.3)**	**0.02**
Red meat					
% EI	8.9 (6.0)	9.40 (6.54)	8.17 (5.21)	−0.4 (−1.2; 0.3)	0.23
Score	0.7 (2.3)	0.84 (2.46)	0.59 (1.93)	−0.1 (−0.2; 0.1)	0.23
Chicken and substitutes					
% EI	7.0 (5.2)	7.36 (5.32)	6.52 (4.92)	0.1 (−0.5; 0.7)	0.73
Score	2.2 (3.2)	2.03 (3.13)	2.35 (3.28)	0.0 (−0.3; 0.4)	0.86
Animal fats					
% EI	3.8 (3.8)	3.23 (3.27)	4.55 (4.37)	0.1 (−0.3; 0.4)	0.72
Score	2.3 (3.9)	2.51 (4.09)	1.92 (3.63)	0.0 (−0.4; 0.5)	0.97
Added sugars					
% EI	11.9 (5.9)	11.65 (6.10)	12.22 (5.70)	−0.3 (−0.8; 0.3)	0.30
Score	0.3 (1.3)	0.38 (1.54)	0.16 (0.74)	−0.1 (−0.2; 0.0)	0.06

PHDI, planetary health diet index; EI (%), percentage of energy intake; SD, standard deviation; DGV, dark green vegetables (ratio of dark green vegetables to total vegetables); ReV, red and orange vegetables (ratio of red and orange vegetables to total vegetables). Linear regression models with per capita household income as continuous predictor and the components of the PHDI as continuous outcome. Robust variance estimates were specified in the models. Adjusted for age, sex, skin color, body fat percentage, and total energy intake/day. ^§^From linear regression model with the Planetary Health Diet Index (PHDI) as continuous outcome and per capita household income per 1 SD (continuous) as predictor. ^‖^Test for linear trend when per capita income were introduced as a continuous predictor in the linear regression. Values in bold (*p* < 0.05).

## 4 Discussion

In this cross-sectional study, our hypothesis was contradicted, as Brazilian children with greater economic vulnerability presented higher PHDI scores. However, this higher relative alignment with the PHDI scoring criteria for economically vulnerable households occurred in a context of overall low adherence to the PHD recommendations, with most of these children living in poverty and experiencing a high prevalence of food insecurity.

It was observed that children from families with income below the poverty line, experiencing moderate or severe FI, receiving government assistance, and living in households with more residents, presented higher adherence to the PHD. However, another Brazilian study demonstrated an inverse association between FI and the PHDI in individuals older than 10 years (30). The findings of our study must be interpreted with caution, as they may reflect specific characteristics of the analyzed sample. Financial constraints imposed by economic vulnerability may be associated with higher scores on the index, since they limit the consumption of higher-cost foods, such as animal-based products, dairy, and fish, while increasing the consumption of more affordable foods, such as legumes and vegetable oils. Although these patterns contributed to a higher PHDI score, they may reflect adaptations to financial scarcity, as shown by the situation of poverty and FI in this sample.

In practical terms, although children in economically vulnerable households may have higher PHDI scores, their diet remain exposed to poorer overall quality. Specifically, children below the poverty line consumed fewer dairy products, fish, fruits, and red and orange vegetables, and more vegetable oils and legumes. Although food expenditures were not assessed in this study, a Brazilian study corroborates these patterns, showing that economically vulnerable families tend to prioritize the acquisition of energy-dense staples such as refined cereals, vegetable oils, legumes, and chicken, while reducing costly items like fruits, vegetables, whole grains, nuts, dairy, and red meat (31). On the other hand, evidence on the relationship between PHDI and overall dietary quality in Brazilian children remains limited; in adults, however, each one-point increase in the PHDI has been associated with better diet quality, reflected as a 0.47-point increase in the Brazilian Healthy Eating Index – Revised (BHEI-R) (7), underscoring that more studies are needed to better understand the applicability of the PHD in contexts of economic vulnerability.

Higher adherence to the PHD observed among economically vulnerable groups may be partially explained by substitution of animal-based foods. For example, beans and rice, a traditional combination of legumes and cereals in the Brazilian dietary pattern, are consumed more frequently by lower-income families than by higher-income families (32). Furthermore, the Brazilian findings are consistent with the Mexican National Health and Nutrition Survey (ENSANUT), which showed that lower food expenditures were associated with greater adherence to the PHD among children and adults, mainly due to reduced consumption of beef, pork, lamb, poultry, dairy products and added sugar (33). In our sample, this substitution pattern, characterized particularly by the frequent consumption of legume-based dishes like rice and beans, although likely driven by financial constraints rather than deliberate health or sustainability choices, results in a dietary pattern that is relatively aligned with the PHD recommendations in economically vulnerable children.

In addition, the relationship of greater economic vulnerability and FI with higher adherence to the PHD can, in part, be explained by the lower consumption of ultra-processed foods (UPF) among vulnerable populations. In a previous study conducted with this same sample, it was observed that children with better socioeconomic conditions had higher UPF consumption (34). Furthermore, a study with Brazilian children and adults showed that individuals in the highest quintile of UPF consumption had lower adherence to the PHD (8). These findings suggest that, in this Brazilian context, higher PHD adherence among the most vulnerable economic population can be simultaneously associated with lower UPF consumption and higher legume intake, resulting in a dietary pattern more consistent with the PHD framework.

Given the challenge of providing healthy food within planetary boundaries for current and future generations (10), child-focused policies, combined with nutrition education, such as school feeding programs, can promote sustainable practices in meal planning and preparation. These include school gardens, recommendations for purchasing sustainable food (organic, local, and seasonal), menu planning, and the reduction of organic and inorganic waste (composting, recycling, donating food, and portion sizes) (35). This perspective on education and promoting lifelong learning opportunities for all is part of Sustainable Development Goal 4 (4, 35), and can contribute to mitigating the effects of financial constraints among economically vulnerable populations while supporting sustainable eating habits, underscoring the need for public policies to prioritize these actions.

Low adherence to the PHD has been reported in children aged 3 to 6 in Finland (36) and Chile (37), as well as in the Global Burden of Disease study, which highlighted regional disparities in the consumption of food groups. While dairy intake exceeds targets in Europe and North America, it remains below recommendations in Latin America and Africa; fruit intake, however, was insufficient across all regions assessed (10, 38). These differences reflect geographic, economic, and cultural factors, emphasizing the need for policies that ensure healthy and sustainable diets, adapted to Brazil’s context and vulnerable population. It should be noted that the PHD defines recommended ranges for food groups based on scientific evidence, aiming to optimize human health, rather than a rigid universal diet, and local adaptation is necessary to align its guidance with regional particularities (10).

This study has some limitations that require caution when generalizing its findings to other populations. The dietary data were self-reported and may be subject to recall bias. The PHDI was validated for Brazilian adults; however, because it considers the relative intake of food groups in relation to the total energy intake, it was calculated proportionally to children’s energy intake. Given the recent adoption of the PHD framework and the scarcity of related studies in childhood, this investigation contributes by highlighting how economic factors can influence dietary patterns that, although aligned with the PHD principles, may also reflect financial constraints on food access. The lack of studies involving children from developing countries underscores the need for further research exploring PHD adherence in different contexts of economic vulnerability, particularly in longitudinal investigations, since the cross-sectional design applied in this study does not allow the establishment of causal relationships between variables. Moreover, it is necessary to validate a specific index for Brazilian children.

Among this study’s strengths, we highlight the use of a photographic food album to estimate portion sizes, as well as the application of three 24-h recalls to obtain more reliable dietary intake data. In addition, the classification of food groups was based on a Brazilian recipe decomposition database, ensuring greater accuracy in the classification of the PHD foods. Furthermore, FI was measured using a scale validated for households with individuals under 18 years old, and the statistical analyses were adjusted for potential confounding factors.

## 5 Conclusion

Although children in economically vulnerable households showed dietary patterns that were relatively more aligned with the PHDI scoring criteria, this alignment appears to be incidental and likely reflects financial constraints due to economic hardship rather than intentional sustainability-oriented choices. Policymakers should interpret this relative alignment with caution and prioritize equitable access to diverse, nutritious foods that support both health and environmental goals. Further studies are needed to clarify how socioeconomic disparities influence proportional alignment with the Planetary Health Diet framework, particularly in low- and middle-income settings.

## Data Availability

The datasets are not publicly available due to confidentiality and controlled access policies. Anonymized data may be obtained from the corresponding author upon reasonable request.
