# Supplementary material for: Associations of economic vulnerability and food insecurity with the Planetary Health Diet in children: PASE study (Brazil)
Source: Front Nutr. 2026 Feb 13;12:1706243. doi: 10.3389/fnut.2025.1706243 (PMC12947699; doi:10.3389/fnut.2025.1706243)
Supplement: Supplementary file 1 [file Table_1.DOCX]

Supplemental Table 1. Components of the Planetary Health Diet Index (PHDI) and criteria for the scoring system (recommended and lower or upper limits recommended*).

| Components | Score range | Recommended value (criteria for maximum score, 10 or 5 points) | Criteria for minimum score (zero points) | Lower and upper limit of recommended intake (cut-off points for gradual scoring) |
| --- | --- | --- | --- | --- |
| **Adequacy components** | |  |  |  |
| Fruits | 0 – 10 | ≥5% of total EI | Zero | – |
| Vegetables | 0 – 10 | ≥3.1% of total EI | Zero | – |
| Nuts and peanuts | 0 – 10 | ≥11.6% of total EI | Zero | – |
| Whole cereals | 0 – 10 | ≥11.3% of total EI | Zero | – |
| Legumes | 0 – 10 | ≥32.4% of total EI | Zero | – |
| **Optimum components** |  |  |  |  |
| Eggs | 0 – 10 | 0.8% of total EI | Zero or >1.5% of total EI | >0.8% to 1.5% of total EI |
| Dairy‡ | 0 – 10 | 6.1% of total EI | Zero or >12.2% of total EI | >6.1% to 12.2% of total EI |
| Tubers | 0 – 10 | 1.6% of total EI | Zero or >3.1% of total EI | >1.6% to 3.1% of total EI |
| Vegetable oils | 0 – 10 | 16.5% of total EI | Zero or >30.7% of total EI | >16.5% to 30.7% of total EI |
| Fish and seafood | 0 – 10 | 1.6% of total EI | Zero or >5.7% of total EI | >1.6% to 5.7% of total EI |
| **Ratio components** |  |  |  |  |
| DGV/total vegetables\|\| | 0 – 5 | 29.5% | Zero | 100% |
| ReV/total vegetables ¶ | 0 – 5 | 38.5% | Zero | 100% |
| **Moderation components** | |  |  |  |
| Red meat** | 0 – 10 | Zero | >2.4% of total EI | >0 to 2.4% of total EI |
| Chicken and substitutes | 0 – 10 | Zero | >5% of total EI | >0 to 5% of total EI |
| Animal fats† | 0 – 10 | Zero | >1.4% of total EI | >0 to 1.4% of total EI |
| Added sugars | 0 - 10 | Zero | >4.8% of total EI | >0 to 4.8% of total EI |

* All values are expressed as caloric ratios between each PHDI component and total daily energy intake (%EI)

‡ Dairy: excluding dairy fats.

|| Dark green vegetables (DGV)/total vegetables: ratio between the energy intake of dark green vegetables (numerator) and the total vegetable energy intake (denominator) multiplied by 100.

¶ Red and orange vegetables (ReV)/total vegetables: ratio between the energy intake of red and orange vegetables (numerator) and the total vegetable energy intake (denominator) multiplied by 100.

** Red meat: including beef and pork.

† Animal fat: lard and dairy fats.

Cacau et al., (2024).
